# Serum 25‐Hydroxyvitamin D Levels and Survival Outcomes in Advanced Biliary Tract Cancer: Results From the NIFTY Trial

**DOI:** 10.1002/cam4.70560

**Published:** 2025-01-03

**Authors:** So Heun Lee, Jaekyung Cheon, Ilhwan Kim, Kyu‐pyo Kim, Baek‐Yeol Ryoo, Jae Ho Jeong, Myoung Joo Kang, Byung Woog Kang, Hyewon Ryu, Ji Sung Lee, Changhoon Yoo

**Affiliations:** ^1^ Department of Oncology, Asan Medical Center University of Ulsan College of Medicine Seoul Korea; ^2^ Division of Hematology‐Oncology Chung‐Ang University Gwangmyeong Hospital Chung‐Ang University College of Medicine Gwangmyeong Korea; ^3^ Division of Oncology, Department of Internal Medicine, Haeundae Paik Hospital Inje University College of Medicine Busan Korea; ^4^ Department of Oncology/Hematology Kyungpook National University Medical Center, Kyungpook National University School of Medicine Daegu Korea; ^5^ Division of Hematology and Oncology, Department of Internal Medicine Chungnam National University Hospital Daejeon Korea; ^6^ Clinical Research Center, Asan Institute for Life Sciences, Asan Medical Center University of Ulsan College of Medicine Seoul Korea; ^7^ Department of Clinical Epidemiology and Biostatics, Asan Medical Center University of Ulsan College of Medicine Seoul Korea

**Keywords:** biliary tract cancer, NIFTY trial, overall survival, vitamin D

## Abstract

**Background:**

Numerous studies have explored the role of vitamin D in various cancers; however, its impact on advanced biliary tract cancers (BTC) within a prospective cohort remains to be investigated. This preplanned subgroup analysis of the NIFTY trial evaluated the prognostic implications of serum vitamin D levels in patients with advanced BTC undergoing second‐line chemotherapy.

**Methods:**

From the 174 patients in the NIFTY trial, a total of 173 patients (99.4%) were included in this analysis comparing a liposomal irinotecan plus 5‐FU/leucovorin group (*n* = 87) and a 5‐FU/leucovorin alone group (*n* = 86). Baseline serum 25‐hydroxyvitamin D (25(OH)D) levels, an indicator of vitamin D status, were analyzed for their association with baseline characteristics and overall survival (OS) in patients undergoing second‐line chemotherapy. Multivariable Cox proportional hazards regression and a restricted cubic spline function were used to assess the association with OS.

**Results:**

There were no significant associations between baseline characteristics and serum 25(OH)D levels. Baseline serum 25(OH)D levels were not associated with OS in either the multivariable Cox proportional hazard regression or restricted cubic spline analysis. In the subgroup analysis, however, higher serum 25(OH)D levels were significantly associated with poorer OS in female patients, while no significant association was observed in male patients, indicating a significant interaction by sex. Additionally, a marginally significant interaction was observed between body mass index and serum 25(OH)D levels for OS, with higher levels associated with better OS in patients who were underweight.

**Conclusions:**

Our preplanned subgroup analysis of the NIFTY trial indicates that the serum 25(OH)D levels did not have a significant effect on OS in the overall patient population with advanced BTC. However, higher serum 25(OH)D levels were associated with worse OS in female patients, underscoring the need for further investigation into the role of vitamin D in BTC.

## 1 Introduction

Biliary tract cancers (BTCs), which include intrahepatic and extrahepatic cholangiocarcinoma as well as gallbladder cancer, represent a heterogeneous spectrum of malignancies [1]. These cancers are rare but aggressive, typically having a median survival of < 1 year [2]. Recent advancements, particularly the addition of programmed cell death 1 (PD‐1) and programmed cell death ligand‐1 (PD‐L1) inhibitors in conjunction with first‐line treatments involving gemcitabine and cisplatin, have improved survival outcomes [3, 4]. However, options for systemic chemotherapy in advanced or metastatic BTC after progression on first‐line chemotherapy remain limited. The NIFTY trial, a multicenter, open‐label, randomized phase 2b study, demonstrated the efficacy and safety of liposomal irinotecan plus 5‐fluorouracil (5‐FU)/leucovorin (LV) compared to 5‐FU/LV alone as a second‐line treatment, reporting a median progression‐free survival (PFS) of 4.2 months versus 1.7 months and median overall survival (OS) of 8.6 months versus 5.3 months [5, 6]. Despite these advances, the overall prognosis of BTC remains poor, underscoring the need for continued exploration of reliable biomarkers and modifiable risk factors to predict survival outcomes [7].

Vitamin D, an essential nutrient and steroidal hormone acquired through ultraviolet radiation exposure and diet, undergoes a two‐step metabolism in the liver and kidneys to synthesize 25‐hydroxyvitamin D [25(OH)D] and 1,25‐dihydroxyvitamin D [1,25(OH)2D], the latter being the most biologically active form of vitamin D [8]. It binds to the vitamin D receptor (VDR), enabling its diverse physiological functions. Beyond its classical role in regulating calcium and bone metabolism, emerging research indicates vitamin D's involvement in multiple cellular processes, including cell proliferation, differentiation, and immunomodulation, all of which may be linked to cancer development and progression [9, 10]. Since Garland et al.'s first prospective study in 1989, which suggested a preventive role for vitamin D in colon cancer [11], its effects have been investigated in various other cancer types. However, no clinical studies have yet examined the prognostic role of vitamin D in a BTC cohort. As a potential prognostic biomarker, we aimed to explore the relationship between baseline vitamin D levels and survival outcomes in patients with advanced BTC who received second‐line systemic treatments of 5‐FU/LV with or without liposomal irinotecan within the prospective framework of the NIFTY trial.

## 2 Methods

### 2.1 Patients and Study Design

This study constitutes a preplanned subgroup analysis of the NIFTY trial, which compared second‐line liposomal irinotecan plus 5‐FU/LV (*n* = 88) with 5‐FU/LV alone (*n* = 86) in patients with advanced BTC. Patients were enrolled between September 5, 2018, and February 18, 2020. Key inclusion criteria of this study were as follows: aged ≥ 19 years; presence of histologically or cytologically confirmed metastatic BTC, including intrahepatic and extrahepatic cholangiocarcinoma and gallbladder cancer; radiologically confirmed disease progression following first‐line treatment with gemcitabine plus cisplatin; an Eastern Cooperative Oncology Group (ECOG) performance status of 0 or 1; and adequate hematological, hepatic, and renal function. Patients with significant gastrointestinal disorders, diarrhea grade ≥ 2, or severe thromboembolic events within the past 6 months were excluded. The dosing schedules of the study treatments and endpoints have been previously described [5].

Correlative analysis of serum 25(OH)D levels for OS was one of the exploratory endpoints. Baseline serum 25(OH)D levels were measured during the screening period at local laboratories. The 25(OH)D is the major circulating form of vitamin D and has a significantly longer half‐life (1000 times longer than that of the active metabolite 1,25(OH)2D), making it the most reliable indicator of vitamin D status [12].

### 2.2 Data Collection and Definitions

Baseline characteristics were sourced from the previously collected database of the NIFTY trial. These included age, sex, body mass index (BMI), and history of previous surgery—factors known to influence serum vitamin D levels based on prior research [13, 14, 15]. Additionally, primary tumor location, metastatic sites, second‐line treatments, and baseline CA 19‐9 levels, which are known to impact survival outcomes, were also recorded [5]. According to the World Health Organization's classification for the Asian population, BMI was categorized as underweight (< 18.5 kg/m^2^), normal (18.5–22.9 kg/m^2^), overweight (23–24.9 kg/m^2^), and obese (≥ 25 kg/m^2^) [16]. Vitamin D deficiency was defined as having serum 25(OH)D levels < 20 ng/mL [17]. The status of tumor progression and survival was retrieved from the updated database of the NIFTY trial [6].

### 2.3 Statistical Analysis

The endpoint of this preplanned subgroup analysis was the correlation between baseline vitamin D levels and OS. OS was defined as the time from randomization in the NIFTY trial to death from any cause. If death was not confirmed before the data cut‐off date, OS was censored at the last date of follow‐up when the patient was known to be alive or at the data cut‐off date, whichever occurred first.

Descriptive statistics were presented as means and standard deviations (SD) for continuous variables and frequencies and percentages for categorical variables. The serum 25(OH)D levels were compared across baseline characteristics using Student's *t*‐test or ANOVA.

The association between serum 25(OH)D levels and OS was examined using a multivariable Cox proportional hazards regression model. This model adjusted for age, sex, ECOG performance status, BMI, primary tumor location, disease status, baseline CA 19‐9 levels, and second‐line treatments, and results were presented as hazard ratios (HR) with 95% confidence intervals (CI). A restricted cubic spline (RCS) function with five knots was used to analyze the relationship between serum 25(OH)D levels and OS, assessing both linear and non‐linear relationships. A serum 25(OH)D level of 20 ng/mL was used as the reference point for calculating adjusted HRs.

Subgroup analyses were performed to evaluate differences in the effects of serum vitamin D on survival outcomes across subgroups known to influence serum 25(OH)D levels. Interaction effects (differences between subgroup‐specific effects) were tested using a two‐sided Wald test in the Cox model. *P*‐values < 0.05 were considered statistically significant. All statistical analyses were conducted using SAS version 9.4 (SAS Institute, Cary, NC, USA) and the R software version 4.2.0 (R Foundation, Vienna, Austria).

## 3 Results

### 3.1 Serum 25(OH)D and Baseline Characteristics

A total of 173 patients (87 in the liposomal irinotecan plus 5‐FU/LV group and 86 in the 5‐FU/LV alone group) were included in this analysis, excluding one patient from the liposomal irinotecan plus 5‐FU/LV group who did not have a measured baseline serum 25(OH)D level. The median age was 64 years (range, 37–84), with 42.8% (*n* = 74) being women. According to the BMI, 40.5% (*n* = 70) of patients were within the normal range, 5.8% (*n* = 10) were categorized as underweight, 23.1% (*n* = 40) as overweight, and 28.9% (*n* = 50) as obese. There were no statistically significant relationships between baseline characteristics and serum 25(OH)D levels (Table 1).

**TABLE 1:** Serum 25‐hydroxyvitamin D levels according to patient baseline characteristics.

		*N* (%)	Serum 25(OH)D		*p*
			Mean	±SD	
Age	< 65	90 (52.0%)	23.2	16.7	0.145
	≥ 65	83 (48.0%)	27.7	23.6	
Sex	Male	99 (57.2%)	24.2	16.5	0.388
	Female	74 (42.8%)	26.9	24.6	
Body mass index†	Underweight	10 (5.8%)	17.5	11.4	0.323
	Normal weight	70 (40.5%)	23.5	16.4	
	Overweight	40 (23.1%)	27.0	18.4	
	Obese	50 (28.9%)	28.5	27.3	
Previous surgery	No	118 (68.2%)	24.3	16.4	0.326
	Yes	55 (31.8%)	27.6	27.0	
ECOG PS	0	38 (22.0%)	24.5	15.8	0.772
	1	135 (78.0%)	25.6	21.5	
Primary tumor location	Intrahepatic	73 (42.2%)	25.7	17.9	0.718
	Extrahepatic	47 (27.2%)	23.2	23.6	
	Gallbladder	53 (30.6%)	26.2	20.2	
Second line treatment	Liposomal irinotecan plus fluorouracil and leucovorin	87 (50.3%)	25.3	21.0	0.994
	Fluorouracil and leucovorin	86 (49.7%)	25.3	19.8	

Abbreviations: 25(OH)D, 25‐hydroxyvitamin D; BMI, body mass index; ECOG PS, Eastern Cooperative Oncology Group performance score; NIH, National Institute of Health; SD, standard deviation; WHO, World Health Organization.

† BMI classification by the NIH and WHO for Asian population. BMI has 3 missing values.

### 3.2 Serum 25(OH)D and OS

During the study period, 155 deaths were recorded, resulting in a median OS of 6.9 months (95% CI, 5.2–8.0) for the overall study cohort. The univariate analysis found no significant association between serum 25(OH)D levels and OS (HR = 1.01 per 10 ng/mL increase; 95% CI, 0.93–1.09; *p* = 0.793; Table S1). In the multivariable Cox regression analysis, which adjusted the impact of key prognostic factors based on the updated dataset of the NIFTY trials [6]—including chemotherapy regimens, metastatic sites, and baseline CA 19‐9 levels—baseline serum 25(OH)D was not statistically associated with OS (HR = 1.06 per 10 ng/mL increase; 95% CI, 0.97–1.16; *p* = 0.197; Table 2).

**TABLE 2:** Multivariable Cox proportional hazards regression for overall survival.

	Adjusted HR (95% CI)	*p*
Treatment		
Liposomal irinotecan plus 5‐FU/LV	1 (Ref)	
5‐FU/LV	1.54 (1.07–2.22)	0.020
Age (per 10 years increase)	1.22 (0.99–1.50)	0.064
Sex		
Male	1 (Ref)	
Female	0.58 (0.41–0.83)	0.003
ECOG PS		
0	1 (Ref)	
1	1.13 (0.73–1.75)	0.573
BMI		
Underweight	1.39 (0.65–2.95)	0.392
Normal weight	1 (Ref)	
Overweight	0.65 (0.41–1.04)	0.075
Obese	0.93 (0.60–1.44)	0.743
Primary tumor location		
Intrahepatic	1 (Ref)	
Extrahepatic	0.88 (0.56–1.40)	0.778
Gallbladder	0.94 (0.61–1.45)	0.590
Disease status		
Recurrent after surgery	1 (Ref)	
Metastatic	0.89 (0.59–1.35)	0.590
Metastatic sites		
Liver metastasis	1.38 (0.92–2.07)	0.117
Lung metastasis	0.85 (0.54–1.34)	0.486
Bone metastasis	1.42 (0.72–2.79)	0.314
Peritoneal seeding	1.52 (1.00–2.32)	0.052
Lymph node metastasis	1.32 (0.90–1.94)	0.151
Soft tissue metastasis	2.20 (0.86–5.63)	0.100
Others	1.42 (0.78–2.58)	0.256
Baseline CA 19‐9 (per 1000 U/mL increase)	1.02 (1.01–1.03)	< 0.001
Baseline serum 25‐hydroxyvitamin D (per 10 ng/mL increase)	1.06 (0.97–1.16)	0.197

Abbreviations: 5‐FU/LV, 5‐fluorouracil/leucovorin; BMI, body mass index; CI, confidence Interval; ECOG PS, Eastern Cooperative Oncology Group performance score; HR, hazard ratio.

**FIGURE 1:**
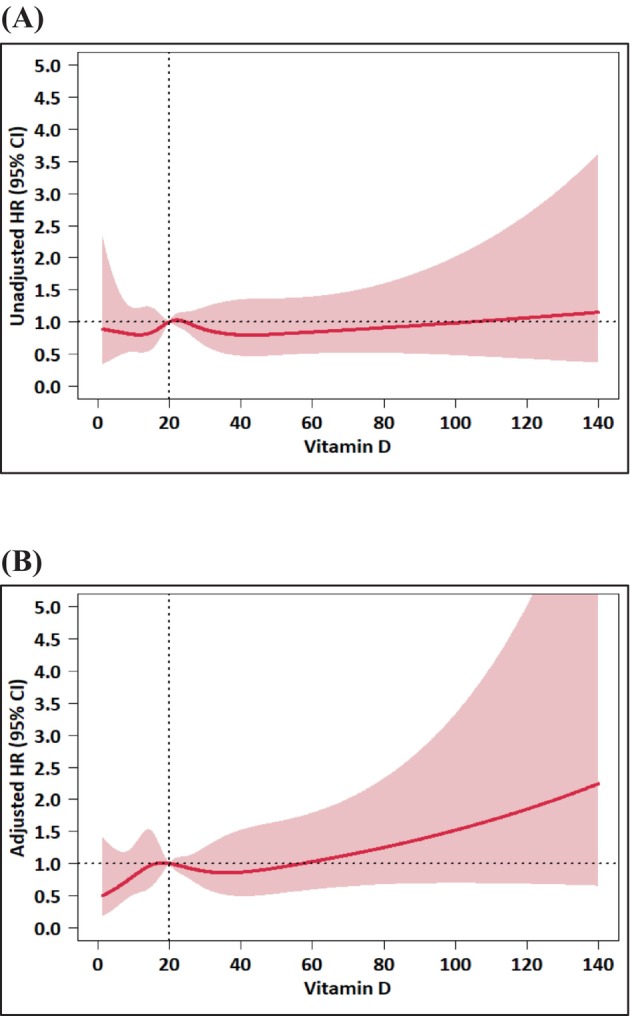
Restricted cubic spline analysis of the association between serum 25‐hydroxyvitamin D levels and overall survival. (A) Unadjusted (B) Adjusted. Adjusted for treatment, age, sex, ECOG PS, BMI, primary tumor location, disease status, metastatic sites (liver, lung, bone, peritoneal seeding, lymph node, soft tissue, others), and baseline CA 19‐9 level. Abbreviations: HR = hazard ratio; CI, confidence Interval; ECOG PS, Eastern Cooperative Oncology Group performance score; BMI, body mass index.

The multivariable RCS regression analysis (Figure 1) also revealed no evidence of either a linear or non‐linear association between serum 25(OH)D levels and OS (*p* = 0.431 for the overall effect and *p* = 0.532 for the non‐linear effect) after adjustment for key prognostic factors.

### 3.3 Subgroup Analyses and Interaction Effect for OS

**FIGURE 2:**
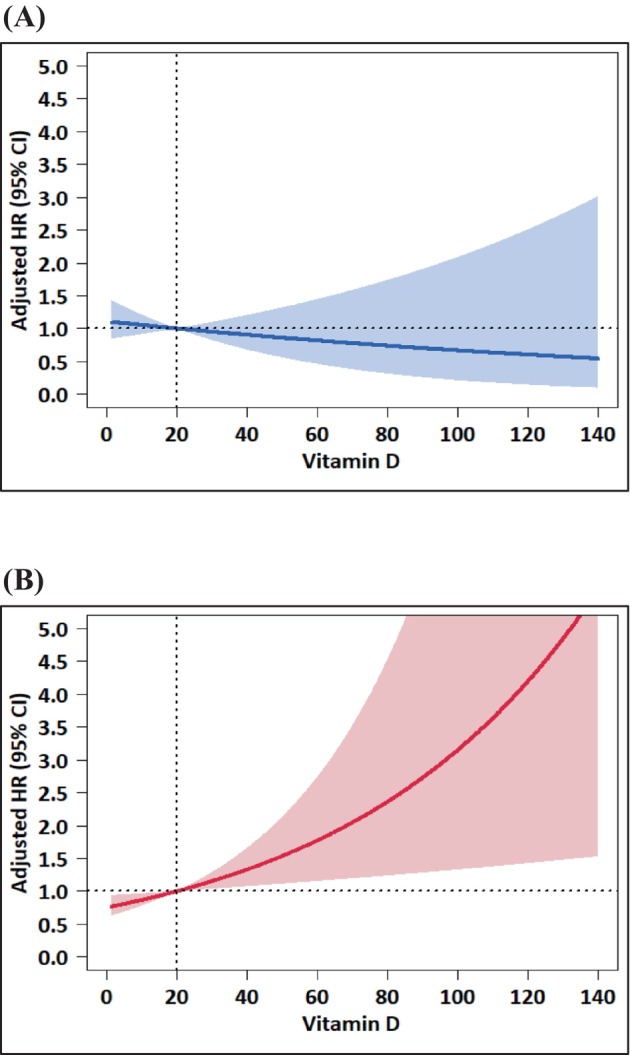
Subgroup analysis of serum 25‐hydroxyvitamin D levels and overall survival stratified by sex. (A) Male (B) Female. Adjusted for treatment, age, ECOG PS, BMI, primary tumor location, disease status, metastatic sites (liver, lung, bone, peritoneal seeding, lymph node, soft tissue, others), and baseline CA 19–9 level. Abbreviations: HR, hazard ratio; CI, confidence Interval; ECOG PS, Eastern Cooperative Oncology Group performance score; BMI, body mass index.

Subgroup analyses were conducted to assess how the relationship between serum 25(OH)D levels and OS varied based on factors such as age, sex, BMI, and previous surgery (Table 3). Notably, higher 25(OH)D levels were significantly associated with worse OS among female patients (HR = 1.15 per 10 ng/mL increase; 95% CI, 1.04–1.28; *p* = 0.009), while no significant association was observed among male patients (HR = 0.94 per 10 ng/mL increase; 95% CI, 0.82–1.08; *p* = 0.390). This indicates a significant interaction by sex (*p* = 0.026), suggesting that the effect of vitamin D on survival outcomes differs depending on sex. This significant sex‐specific difference in the association between serum 25(OH)D levels and OS was further illustrated in the RCS analysis (Figure 2).

**TABLE 3:** Subgroup analysis of the interaction effect on overall survival.

	Unadjusted			Adjusted		
	HR (95% CI)	*p*	*p* for interaction	HR (95% CI)	*p*	*p* for interaction
Age			0.509			0.667
< 65	0.97 (0.85–1.11)	0.702		1.03 (0.89–1.19)	0.687	
≥ 65	1.03 (0.94–1.13)	0.552		1.07 (0.97–1.19)	0.191	
Sex			0.232			0.026
Male	0.96 (0.84–1.09)	0.503		0.94 (0.82–1.08)	0.390	
Female	1.06 (0.96–1.16)	0.270		1.15 (1.04–1.28)	0.009	
BMI			0.167			0.059
Underweight	0.48 (0.25–0.93)	0.029		0.49 (0.25–0.93)	0.030	
Normal weight	1.02 (0.89–1.17)	0.774		1.15 (0.98–1.36)	0.082	
Overweight	1.01 (0.85–1.21)	0.882		0.98 (0.82–1.19)	0.869	
Obese	1.03 (0.93–1.15)	0.546		1.10 (0.98–1.24)	0.099	
Previous surgery			0.831			0.443
No	1.00 (0.90–1.12)	0.944		1.02 (0.90–1.15)	0.773	
Yes	1.02 (0.91–1.14)	0.711		1.09 (0.97–1.22)	0.163	

*Note:* HR for baseline 25‐hydroxyvitamin D level (per 10 ng/mL increase). Adjusted for treatment, age, ECOG PS, primary tumor location, disease status, metastatic sites (liver, lung, bone, peritoneal seeding, lymph node, soft tissue, others), BMI, and baseline CA 19‐9 level.

Abbreviations: 25(OH)D, 25‐hydroxyvitamin D; BMI, body mass index; CI, confidence interval; ECOG PS, Eastern Cooperative Oncology Group performance score; HR, hazard ratio.

There was a marginally significant interaction between BMI and serum 25(OH)D levels regarding OS (*p* = 0.059, Table 3) in the overall patient cohort. However, in the subgroup of patients who were underweight (BMI < 18.5 kg/m^2^), higher 25(OH)D levels were associated with better OS (HR = 0.49 per 10 ng/mL increase; 95% CI, 0.25–0.93; *p* = 0.030). No significant interactions were found across the subgroups of age (*p* = 0.667) or history of previous surgery (*p* = 0.443).

## 4 Discussion

In this preplanned subgroup analysis of the NIFTY randomized clinical trial, which investigated the role of vitamin D, serum 25(OH)D levels were not associated with survival outcomes in patients with advanced BTC treated with second‐line cytotoxic chemotherapy. However, higher 25(OH)D levels were significantly associated with worse survival among female patients, suggesting that the impact of serum vitamin D levels on survival outcomes may vary based on the patient's sex.

Recent meta‐analyses have highlighted an inverse association between vitamin D levels and colorectal cancer incidence, showing a 20%–39% reduction in risk [18, 19]. These beneficial effects have also been confirmed in cancer prognosis and mortality, particularly in advanced or metastatic cancer cohorts [20, 21, 22, 23, 24]. Higher vitamin D levels or supplementation have been associated with significant improvements in adverse outcomes in colon cancer, as evidenced by reduced HRs for PFS, recurrence, or mortality, with reductions ranging from 12% to 32% [22, 23, 24, 25, 26]. While the most convincing data exist for colorectal cancers, studies on other cancer types generally show inverse or nonsignificant relationships between vitamin D levels and cancer outcomes, except for prostate cancer, where elevated vitamin D levels are linked to an increased risk for its incidence [27, 28, 29]. In breast cancer, associations have been observed in specific subtypes, such as triple‐negative breast cancer [30]. Despite ongoing research and the accumulation of data across various cancer types, the relationship between vitamin D levels and BTC outcomes has not been explored.

While clinical exploration of this relationship in BTC is limited, several in vitro and in vivo studies have been conducted. VDR, the exclusive mediator of 1,25(OH)_2_D effects, exhibits increased expression during cancer development in cholangiocarcinoma cells [31, 32]. Administration of 1,25(OH)_2_D in vitro has been shown to reduce cell proliferation, especially in cells with higher VDR expression. This treatment also led to a 2‐ to 10‐fold increase in VDR binding events, making the cells more responsive to the effects of 1,25(OH)_2_D, which was associated with a better prognosis [31]. Additionally, it altered gene expression profiles related to cell cycle regulation, such as the downregulation of *lipocalin 2 (LCN2)* and decreased expression of *CYP24A1*, a vitamin D‐degrading enzyme [33]. These findings suggest that vitamin D may inhibit tumor growth and progression in BTC.

To the best of our knowledge, this is the first study to investigate the implications of serum 25(OH)D in patients with advanced BTC based on a prospective trial. Our study did not find a significant association between serum 25(OH)D levels and OS in the overall patient population with advanced BTC treated with second‐line chemotherapy. However, subgroup analyses suggested potential sex‐specific variations in the clinical impact of vitamin D on OS. In female patients, worse OS was associated with higher serum 25(OH)D levels. These sex‐specific variations in the impact of vitamin D have also been suggested in previous studies on colorectal cancer [18, 19]. In a pooled analysis of 17 international colorectal cancer cohorts, elevated serum 25(OH)D levels were significantly associated with a lower risk of colorectal cancer, particularly in women (relative risks [RR] per 25 nmol/L increase, with a 19% risk reduction for women vs. a 7% risk reduction for men; two‐sided *p*‐value for interaction = 0.008) [19]. The sex‐specific impact of vitamin D on patient survival might be explained by the findings that more genes are affected by vitamin D in females than in males (approximately three times), and sex hormones contribute to the expression of genes related to vitamin D [34, 35]. However, these findings are yet to be fully understood in terms of their mechanisms. Furthermore, the impact of higher serum 25(OH)D levels in the female patients of our study cohort was contrary to the findings of previous studies on colorectal cancer, although it is difficult to directly compare our study outcomes with those of previous studies owing to different tumor types and clinical settings. These observations underscore the need for further studies to provide mechanistic insights into how vitamin D affects patients with advanced cancer and its potential sex‐specific effects.

The interaction between BMI and serum 25(OH)D levels was marginally significant in our study. In the subgroup of patients who were underweight, higher 25(OH)D levels were associated with better OS. Our findings are supported in part by the results of the VITamin D and OmegA‐3 (VITAL) trial, which showed that the effect size of vitamin D treatment on cancer mortality progressively decreased with increasing BMI (BMI < 25: HR 0.58, *p* = 0.007; BMI 25–30: HR 0.89, *p* = 0.472; BMI ≥ 30: HR 1.15, *p* = 0.518; *p*‐value for interaction = 0.02) [20]. This might be explained in part by factors such as decreased bioavailability and volumetric dilution of vitamin D [36, 37]. However, this finding should be validated in studies with large sample sizes, considering that the number of patients who were underweight in this study was too small (*n* = 10).

Although the current analysis has notable strengths, such as a subgroup analysis of a prospective multicenter trial with closely monitored clinical outcomes, several limitations exist in our study. This study is based on a single baseline measurement of vitamin D levels, and its seasonal variability was not considered in the analysis. These might limit the comprehensiveness of the assessment of the impact of vitamin D levels on clinical outcomes. As our study population was limited to patients with second‐line palliative chemotherapy, our findings do not preclude the potential implications of serum vitamin D levels in those with earlier stages of the disease. Furthermore, limited generalizability, particularly for non‐Asian ethnicities, and the lack of consideration of molecular alterations represents potential limitations in the interpretation of our findings.

## 5 Conclusions

In conclusion, our preplanned analysis of the NIFTY trial found no significant association between serum 25(OH)D levels and OS in patients with advanced BTC treated with second‐line chemotherapy. However, subgroup analyses revealed a significant association between serum 25(OH)D levels and OS in female patients. These findings underscore the need for further research on the role of vitamin D in patients with BTC.

## Author Contributions

**So Heun Lee:** data curation (equal), formal analysis (equal), investigation (equal), visualization (equal), writing – original draft (equal), writing – review and editing (equal). **Jaekyung Cheon:** data curation (equal), formal analysis (equal), investigation (equal), visualization (equal), writing – original draft (equal), writing – review and editing (equal). **Ilhwan Kim:** data curation (equal), formal analysis (equal), writing – review and editing (equal). **Kyu‐pyo Kim:** data curation (equal), formal analysis (equal), writing – review and editing (equal). **Baek‐Yeol Ryoo:** data curation (equal), formal analysis (equal), writing – review and editing (equal). **Jae Ho Jeong:** data curation (equal), formal analysis (equal), writing – review and editing (equal). **Myoung Joo Kang:** data curation (equal), Formal analysis (equal), Writing – review and editing (equal). **Byung Woog Kang:** data curation (equal), Formal analysis (equal), writing – review and editing (equal). **Hyewon Ryu:** data curation (equal), formal analysis (equal), writing – review and editing (equal). **Ji Sung Lee:** conceptualization (equal), data curation (equal), formal analysis (equal), investigation (equal), methodology (equal), supervision (equal), validation (equal), writing – review and editing (equal). **Changhoon Yoo:** conceptualization (equal), data curation (equal), formal analysis (equal), funding acquisition (equal), investigation (equal), supervision (equal), writing – review and editing (equal).

## Disclosure

The funders had no role in the study design, data collection, data management, data analysis, data interpretation, or the writing of the report.

## Ethics Statement

This study was approved by the ethics committee of Asan Medical Center (IRB No. 20180232), ensuring that all procedures met the required ethical standards.

## Consent

Informed consent was obtained from all patients before their participation in the study, which was conducted in accordance with the Declaration of Helsinki.

## Conflicts of Interest

The authors declare no conflicts of interest.

## Supporting information


**Data S1.** Supporting Information.

## Data Availability

The data that support the findings of this study are available from the corresponding author upon reasonable request. However, access to these data is restricted and will not be publicly available due to privacy concerns.

## References

[1] C. Yoo , J. Hyung , and S. L. Chan , “Recent Advances in Systemic Therapy for Advanced Intrahepatic Cholangiocarcinoma,” Liver Cancer 13 (2023): 1–1, 135. 38638168 10.1159/000531458 PMC11023692

[2] A. Lamarca , J. Edeline , M. G. McNamara , et al., “Current Standards and Future Perspectives in Adjuvant Treatment for Biliary Tract Cancers,” Cancer Treatment Reviews 84 (2020): 101936, 10.1016/j.ctrv.2019.101936. 31986437

[3] D. Y. Oh , A. Ruth He , S. Qin , et al., “Durvalumab Plus Gemcitabine and Cisplatin in Advanced Biliary Tract Cancer,” NEJM Evidence 1, no. 8 (2022): 1–11, 10.1056/EVIDoa2200015. 38319896

[4] R. K. Kelley , M. Ueno , C. Yoo , et al., “Pembrolizumab in Combination With Gemcitabine and Cisplatin Compared With Gemcitabine and Cisplatin Alone for Patients With Advanced Biliary Tract Cancer (KEYNOTE‐966): A Randomised, Double‐Blind, Placebo‐Controlled, Phase 3 Trial,” Lancet 401, no. 10391 (2023): 1853–1865, 10.1016/S0140-6736(23)00727-4. 37075781

[5] C. Yoo , K. P. Kim , J. H. Jeong , et al., “Liposomal Irinotecan Plus Fluorouracil and Leucovorin Versus Fluorouracil and Leucovorin for Metastatic Biliary Tract Cancer After Progression on Gemcitabine Plus Cisplatin (NIFTY): A Multicentre, Open‐Label, Randomised, Phase 2b Study,” Lancet Oncology 22, no. 11 (2021): 1560–1572, 10.1016/S1470-2045(21)00486-1. 34656226

[6] J. Hyung , I. Kim , K. P. Kim , et al., “Treatment With Liposomal Irinotecan Plus Fluorouracil and Leucovorin for Patients With Previously Treated Metastatic Biliary Tract Cancer: The Phase 2b NIFTY Randomized Clinical Trial,” JAMA Oncology 9, no. 5 (2023): 692–699, 10.1001/jamaoncol.2023.0016. 36951834 PMC10037199

[7] Y. H. Bang , C.‐k. Lee , K. Bang , et al., “Artificial Intelligence‐Powered Spatial Analysis of Tumor‐Infiltrating Lymphocytes as a Potential Biomarker for Immune Checkpoint Inhibitors in Patients With Biliary Tract Cancer,” Clinical Cancer Research 30, no. 20 (2024): 4635–4643. 39150517 10.1158/1078-0432.CCR-24-1265

[8] G. Jones , D. E. Prosser , and M. Kaufmann , “Cytochrome P450‐Mediated Metabolism of Vitamin D,” Journal of Lipid Research 55, no. 1 (2014): 13–31, 10.1194/jlr.R031534. 23564710 PMC3927478

[9] D. Feldman , A. V. Krishnan , S. Swami , E. Giovannucci , and B. J. Feldman , “The Role of Vitamin D in Reducing Cancer Risk and Progression,” Nature Reviews. Cancer 14, no. 5 (2014): 342–357, 10.1038/nrc3691. 24705652

[10] C. Carlberg and A. Munoz , “An Update on Vitamin D Signaling and Cancer,” Seminars in Cancer Biology 79 (2022): 217–230, 10.1016/j.semcancer.2020.05.018. 32485310

[11] C. F. Garland , G. W. Comstock , F. C. Garland , K. J. Helsing , E. K. Shaw , and E. D. Gorham , “Serum 25‐Hydroxyvitamin D and Colon Cancer: Eight‐Year Prospective Study,” Lancet 2, no. 8673 (1989): 1176–1178, 10.1016/s0140-6736(89)91789-3. 2572900

[12] K. D. Cashman , E. G. van den Heuvel , R. J. Schoemaker , D. P. Prévéraud , H. M. Macdonald , and J. Arcot , “25‐Hydroxyvitamin D as a Biomarker of Vitamin D Status and Its Modeling to Inform Strategies for Prevention of Vitamin D Deficiency Within the Population,” Advances in Nutrition 8, no. 6 (2017): 947–957, 10.3945/an.117.015578. 29141976 PMC5682995

[13] G. Muscogiuri , L. Barrea , C. D. Somma , et al., “Sex Differences of Vitamin D Status Across BMI Classes: An Observational Prospective Cohort Study,” Nutrients 11, no. 12 (2019): 3034, 10.3390/nu11123034. 31842281 PMC6950363

[14] D. D. Bikle , “Vitamin D Insufficiency/Deficiency in Gastrointestinal Disorders,” Journal of Bone and Mineral Research 22, no. S2 (2007): V50–V54, 10.1359/jbmr.07s208. 18290722

[15] E. Giovannucci , Y. Liu , E. B. Rimm , et al., “Prospective Study of Predictors of Vitamin D Status and Cancer Incidence and Mortality in Men,” Journal of the National Cancer Institute 98, no. 7 (2006): 451–459, 10.1093/jnci/djj101. 16595781

[16] World Health Organization, Regional Office for the Western Pacific , The Asia‐Pacific Perspective: Redefining Obesity and Its Treatment (Sydney: Health Communications Australia, 2000), 55.

[17] M. F. Holick , N. C. Binkley , H. A. Bischoff‐Ferrari , et al., “Evaluation, Treatment, and Prevention of Vitamin D Deficiency: An Endocrine Society Clinical Practice Guideline,” Journal of Clinical Endocrinology and Metabolism 96, no. 7 (2011): 1911–1930, 10.1210/jc.2011-0385. 21646368

[18] P. Hernandez‐Alonso , H. Boughanem , S. Canudas , et al., “Circulating Vitamin D Levels and Colorectal Cancer Risk: A Meta‐Analysis and Systematic Review of Case‐Control and Prospective Cohort Studies,” Critical Reviews in Food Science and Nutrition 63, no. 1 (2023): 1–17, 10.1080/10408398.2021.1939649. 34224246

[19] M. L. McCullough , E. S. Zoltick , S. J. Weinstein , et al., “Circulating Vitamin D and Colorectal Cancer Risk: An International Pooling Project of 17 Cohorts,” Journal of the National Cancer Institute 111, no. 2 (2019): 158–169, 10.1093/jnci/djy087. 29912394 PMC6376911

[20] P. D. Chandler , W. Y. Chen , O. N. Ajala , et al., “Effect of Vitamin D3 Supplements on Development of Advanced Cancer: A Secondary Analysis of the VITAL Randomized Clinical Trial,” JAMA Network Open 3, no. 11 (2020): e2025850, 10.1001/jamanetworkopen.2020.25850. 33206192 PMC7675103

[21] K. Ng , H. S. Nimeiri , N. J. McCleary , et al., “Effect of High‐Dose vs Standard‐Dose Vitamin D3 Supplementation on Progression‐Free Survival Among Patients With Advanced or Metastatic Colorectal Cancer: The SUNSHINE Randomized Clinical Trial,” Journal of the American Medical Association 321, no. 14 (2019): 1370–1379, 10.1001/jama.2019.2402. 30964527 PMC6459117

[22] M. A. Fuchs , C. Yuan , K. Sato , et al., “Predicted Vitamin D Status and Colon Cancer Recurrence and Mortality in CALGB 89803 (Alliance),” Annals of Oncology 28, no. 6 (2017): 1359–1367, 10.1093/annonc/mdx109. 28327908 PMC5789809

[23] A. Ottaiano , M. L. Iacovino , M. Santorsola , et al., “Circulating Vitamin D Level Before Initiating Chemotherapy Impacts on the Time‐To‐Outcome in Metastatic Colorectal Cancer Patients: Systematic Review and Meta‐Analysis,” Journal of Translational Medicine 22, no. 1 (2024): 119, 10.1186/s12967-024-04889-2. 38291479 PMC10826188

[24] A. Ottaiano , S. Facchini , M. Santorsola , et al., “Circulating Vitamin D Level and Its Impact on Mortality and Recurrence in Stage III Colorectal Cancer Patients: A Systematic Review and Meta‐Analysis,” Cancers 15, no. 11 (2023): 3012, 10.3390/cancers15113012. 37296974 PMC10251929

[25] P. G. Vaughan‐Shaw , L. F. Buijs , J. P. Blackmur , et al., “The Effect of Vitamin D Supplementation on Survival in Patients With Colorectal Cancer: Systematic Review and Meta‐Analysis of Randomised Controlled Trials,” British Journal of Cancer 123, no. 11 (2020): 1705–1712, 10.1038/s41416-020-01060-8. 32929196 PMC7686489

[26] R. Chowdhury , S. Kunutsor , A. Vitezova , et al., “Vitamin D and Risk of Cause Specific Death: Systematic Review and Meta‐Analysis of Observational Cohort and Randomised Intervention Studies,” BMJ 348 (2014): g1903, 10.1136/bmj.g1903. 24690623 PMC3972416

[27] J. Gao , W. Wei , G. Wang , H. Zhou , Y. Fu , and N. Liu , “Circulating Vitamin D Concentration and Risk of Prostate Cancer: A Dose‐Response Meta‐Analysis of Prospective Studies,” Therapeutics and Clinical Risk Management 14 (2018): 95–104, 10.2147/TCRM.S149325. 29386901 PMC5767091

[28] X. Zhang , X. Z. Huang , W. J. Chen , et al., “Plasma 25‐Hydroxyvitamin D Levels, Vitamin D Intake, and Pancreatic Cancer Risk or Mortality: A Meta‐Analysis,” Oncotarget 8, no. 38 (2017): 64395–64406, 10.18632/oncotarget.18888. 28969079 PMC5610011

[29] Q. Feng , H. Zhang , Z. Dong , Y. Zhou , and J. Ma , “Circulating 25‐Hydroxyvitamin D and Lung Cancer Risk and Survival: A Dose‐Response Meta‐Analysis of Prospective Cohort Studies,” Medicine 96, no. 45 (2017): e8613, 10.1097/MD.0000000000008613. 29137092 PMC5690785

[30] S. Hossain , M. A. Beydoun , H. A. Beydoun , X. Chen , A. B. Zonderman , and R. J. Wood , “Vitamin D and Breast Cancer: A Systematic Review and Meta‐Analysis of Observational Studies,” Clinical Nutrition ESPEN 30 (2019): 170–184, 10.1016/j.clnesp.2018.12.085. 30904218 PMC6570818

[31] W. Seubwai , C. Wongkham , A. Puapairoj , N. Khuntikeo , and S. Wongkham , “Overexpression of Vitamin D Receptor Indicates a Good Prognosis for Cholangiocarcinoma: Implications for Therapeutics,” Cancer 109, no. 12 (2007): 2497–2505, 10.1002/cncr.22716. 17487855

[32] K. C. Chiang , C. N. Yeh , K. J. Lin , et al., “Chemopreventive and Chemotherapeutic Effect of Dietary Supplementation of Vitamin D on Cholangiocarcinoma in a Chemical‐Induced Animal Model,” Oncotarget 5, no. 11 (2014): 3849–3861, 10.18632/oncotarget.2000. 24939880 PMC4116525

[33] L. Kennedy , K. Baker , K. Hodges , et al., “Dysregulation of Vitamin D3 Synthesis Leads to Enhanced Cholangiocarcinoma Growth,” Digestive and Liver Disease 45, no. 4 (2013): 316–322, 10.1016/j.dld.2012.12.012. 23375797

[34] P. Protiva , H. S. Cross , M. E. Hopkins , et al., “Chemoprevention of Colorectal Neoplasia by Estrogen: Potential Role of Vitamin D Activity,” Cancer Prevention Research 2, no. 1 (2009): 43–51, 10.1158/1940-6207.CAPR-08-0103. 19139017

[35] X. Garcia‐Albeniz , A. Rudolph , C. Hutter , et al., “CYP24A1 Variant Modifies the Association Between Use of Oestrogen Plus Progestogen Therapy and Colorectal Cancer Risk,” British Journal of Cancer 114, no. 2 (2016): 221–229, 10.1038/bjc.2015.443. 26766742 PMC4815813

[36] A. T. Drincic , L. A. Armas , E. E. Van Diest , and R. P. Heaney , “Volumetric Dilution, Rather Than Sequestration Best Explains the Low Vitamin D Status of Obesity,” Obesity 20, no. 7 (2012): 1444–1448, 10.1038/oby.2011.404. 22262154

[37] J. Wortsman , L. Y. Matsuoka , T. C. Chen , Z. Lu , and M. F. Holick , “Decreased Bioavailability of Vitamin D in Obesity,” American Journal of Clinical Nutrition 72, no. 3 (2000): 690–693, 10.1093/ajcn/72.3.690. 10966885

