# Supplementary material for: Serum 25‐Hydroxyvitamin D Levels and Survival Outcomes in Advanced Biliary Tract Cancer: Results From the NIFTY Trial
Source: Cancer Med. 2025 Jan 3;14(1):e70560. doi: 10.1002/cam4.70560 (PMC11696253; doi:10.1002/cam4.70560)
Supplement: Supplementary file 1 — Data S1. Supporting Information. [file CAM4-14-e70560-s001.docx]

**Supplementary Table 1. Univariate and Multivariate Cox Proportional Hazards Regression for Overall Survival**

|  | **Unadjusted** | | **Adjusted** | |
| --- | --- | --- | --- | --- |
|  | **HR (95% CI)** | **P value** | **HR (95% CI)** | **P value** |
| **Treatment** |  |  |  |  |
| Liposomal irinotecan plus 5-FU/LV | 1 (Ref) |  | 1 (Ref) |  |
| 5-FU/LV | 1.50 (1.09-2.07) | 0.012 | 1.54 (1.07-2.22) | 0.020 |
| **Age**  **(per 10 years increase)** | 1.12 (0.94-1.33) | 0.200 | 1.22 (0.99-1.50) | 0.064 |
| **Sex** |  |  |  |  |
| Male | 1 (Ref) |  | 1 (Ref) |  |
| Female | 0.67 (0.49-0.93) | 0.017 | 0.58 (0.41-0.83) | 0.003 |
| **ECOG PS** |  |  |  |  |
| 0 | 1 (Ref) |  | 1 (Ref) |  |
| 1 | 1.22 (0.83-1.80) | 0.309 | 1.13 (0.73-1.75) | 0.573 |
| **BMI** |  |  |  |  |
| Underweight | 2.10 (1.07-4.13) | 0.031 | 1.39 (0.65-2.95) | 0.392 |
| Normal weight | 1 (Ref) |  | 1 (Ref) |  |
| Overweight | 0.81 (0.53-1.23) | 0.330 | 0.65 (0.41-1.04) | 0.075 |
| Obese | 1.01 (0.69-1.49) | 0.940 | 0.93 (0.60-1.44) | 0.743 |
| **Primary tumor location** |  |  |  |  |
| Intrahepatic | 1 (Ref) |  | 1 (Ref) |  |
| Extrahepatic | 0.88 (0.60-1.29) | 0.516 | 0.88 (0.56-1.40) | 0.778 |
| Gallbladder | 0.86 (0.59-1.26) | 0.442 | 0.94 (0.61-1.45) | 0.590 |
| **Disease status** |  |  |  |  |
| Recurrent after surgery | 1 (Ref) |  | 1 (Ref) |  |
| Metastatic | 0.87 (0.62-1.22) | 0.426 | 0.89 (0.59-1.35) | 0.590 |
| **Metastatic sites** |  |  |  |  |
| Liver metastasis | 1.20 (0.85-1.70) | 0.306 | 1.38 (0.92-2.07) | 0.117 |
| Lung metastasis | 0.86 (0.58-1.27) | 0.440 | 0.85 (0.54-1.34) | 0.486 |
| Bone metastasis | 1.19 (0.66-2.15) | 0.565 | 1.42 (0.72-2.79) | 0.314 |
| Peritoneal seeding | 1.17 (0.81-1.68) | 0.410 | 1.52 (1.00-2.32) | 0.052 |
| Lymph node metastasis | 1.23 (0.89-1.70) | 0.206 | 1.32 (0.90-1.94) | 0.151 |
| Soft tissue metastasis | 1.31 (0.58-2.96) | 0.522 | 2.20 (0.86-5.63) | 0.100 |
| Others | 1.22 (0.71-2.09) | 0.464 | 1.42 (0.78-2.58) | 0.256 |
| **Baseline CA 19-9 (per 1000 U/mL increase)** | 1.02 (1.01-1.02) | <0.001 | 1.02 (1.01-1.03) | <0.001 |
| **Baseline serum 25-hydroxyvitamin D**  **(per 10ng/mL increase)** | 1.01 (0.93-1.09) | 0.793 | 1.06 (0.97-1.16) | 0.197 |

Abbreviations: HR=Hazard ratio; CI=Confidence Interval; 5-FU/LV=5-fluorouracil/leucovorin; ECOG PS=Eastern Cooperative Oncology Group performance score; BMI=Body mass index.

**Supplementary Figure 1. Subgroup Analysis of Serum 25-hydroxyvitamin D Levels and Overall Survival Stratified by BMI.** (A) Underweight (B) Normal (C) Overweight (D) Obese.

**(A)**


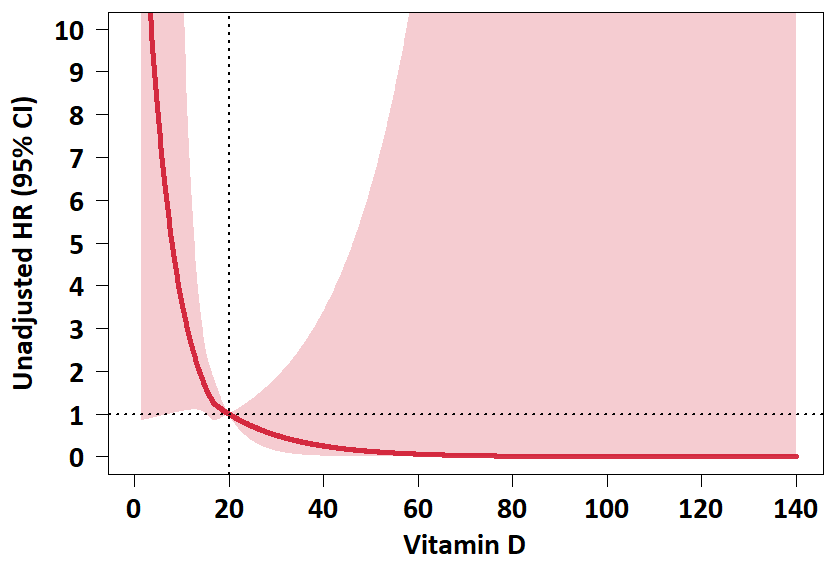


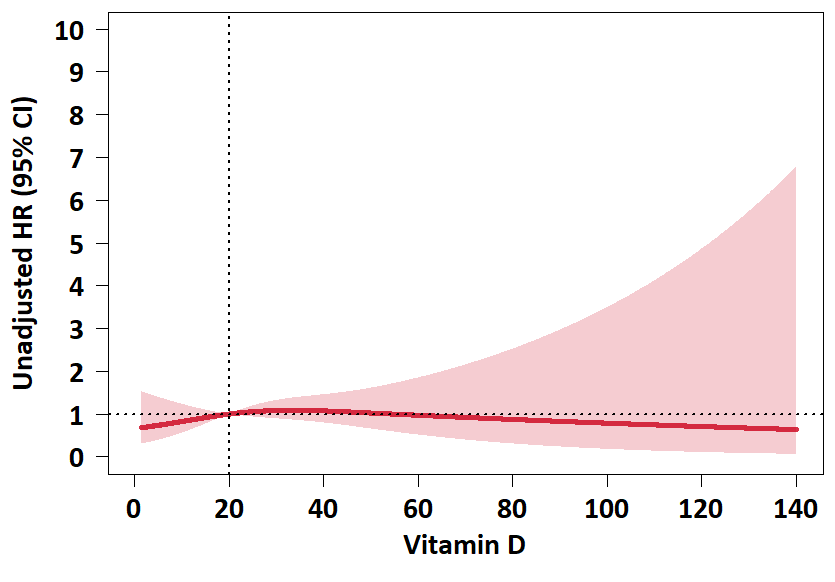
**(B)**


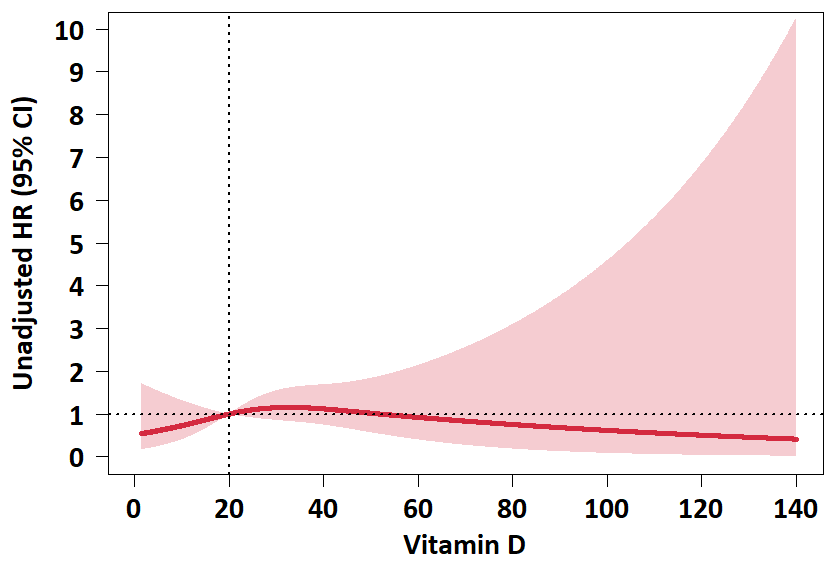
**(C)**


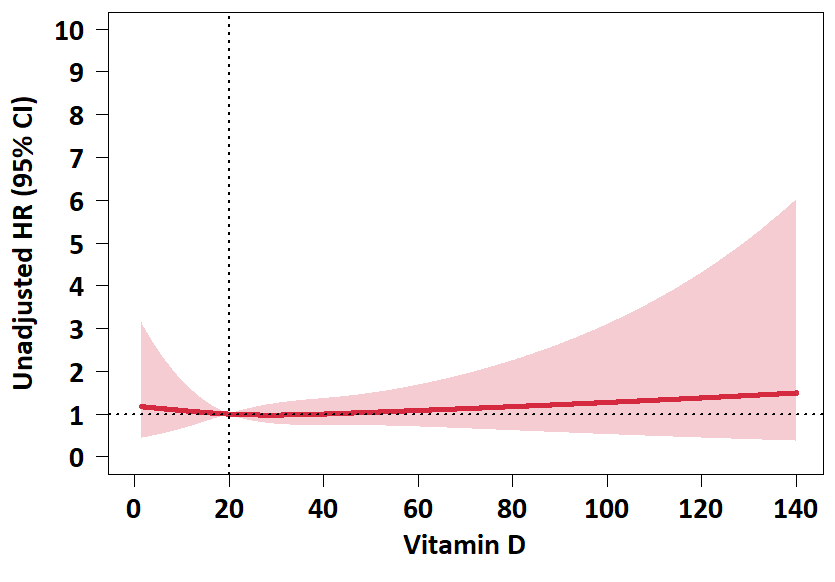
**(D)**

Adjusted for Treatment, Age, ECOG PS, Sex, Primary tumor location, Disease status, Metastatic sites (liver, lung, bone, peritoneal seeding, lymph node, soft tissue, others), and Baseline CA 19-9 level.

Abbreviations: BMI=Body mass index; HR=Hazard ratio; CI=Confidence Interval; ECOG PS=Eastern Cooperative Oncology Group performance score.
